# Supplementary material for: COZOID: contact zone identifier for visual analysis of protein-protein interactions
Source: BMC Bioinformatics. 2018 Apr 6;19:125. doi: 10.1186/s12859-018-2113-6 (PMC5889581; doi:10.1186/s12859-018-2113-6)
Supplement: Supplementary file 2 — Software build. Executable binary file of the software tool. (ZIP 118,784 kb) [file 12859_2018_2113_MOESM2_ESM.zip › cozoid/java/docs/beansbinding-1.2.1-doc.zip!/org/jdesktop/el/impl/parser/class-use/Node.html]

Uses of Interface org.jdesktop.el.impl.parser.Node (Beans Binding)


---


|  |  |  |
| --- | --- | --- |
| **Overview** \| **Package** \| **Class** \| **Use** \| **Tree** \| **Deprecated** \| **Index** \| **Help** | |  |
| PREV   NEXT | **FRAMES**    **NO FRAMES**     **All Classes** |  |


---


## **Uses of Interface org.jdesktop.el.impl.parser.Node**

| Packages that use Node | |
| --- | --- |
| **org.jdesktop.el.impl** | Customized version of EL for Beans Binding; not for general use. |
| **org.jdesktop.el.impl.lang** | Customized version of EL for Beans Binding; not for general use. |
| **org.jdesktop.el.impl.parser** | Customized version of EL for Beans Binding; not for general use. |

Uses of Node in org.jdesktop.el.impl

Constructors in org.jdesktop.el.impl with parameters of type Node

`MethodExpressionImpl(java.lang.String expr,
Node node,
FunctionMapper fnMapper,
VariableMapper varMapper,
java.lang.Class expectedType,
java.lang.Class[] paramTypes)`

`ValueExpressionImpl(java.lang.String expr,
Node node,
FunctionMapper fnMapper,
VariableMapper varMapper,
java.lang.Class expectedType)`

Uses of Node in org.jdesktop.el.impl.lang

| Methods in org.jdesktop.el.impl.lang that return Node | |
| --- | --- |
| `static Node` | `ExpressionBuilder.createNode(java.lang.String expr)` |

| Methods in org.jdesktop.el.impl.lang with parameters of type Node | |
| --- | --- |
| `void` | `ExpressionBuilder.visit(Node node)` |

Uses of Node in org.jdesktop.el.impl.parser

| Classes in org.jdesktop.el.impl.parser that implement Node | |
| --- | --- |
| `class` | `ArithmeticNode` |
| `class` | `AstAnd` |
| `class` | `AstBracketSuffix` |
| `class` | `AstChoice` |
| `class` | `AstCompositeExpression` |
| `class` | `AstDeferredExpression` |
| `class` | `AstDiv` |
| `class` | `AstDotSuffix` |
| `class` | `AstDynamicExpression` |
| `class` | `AstEmpty` |
| `class` | `AstEqual` |
| `class` | `AstFalse` |
| `class` | `AstFloatingPoint` |
| `class` | `AstFunction` |
| `class` | `AstGreaterThan` |
| `class` | `AstGreaterThanEqual` |
| `class` | `AstIdentifier` |
| `class` | `AstInteger` |
| `class` | `AstLessThan` |
| `class` | `AstLessThanEqual` |
| `class` | `AstLiteralExpression` |
| `class` | `AstMinus` |
| `class` | `AstMod` |
| `class` | `AstMult` |
| `class` | `AstNegative` |
| `class` | `AstNot` |
| `class` | `AstNotEqual` |
| `class` | `AstNull` |
| `class` | `AstOr` |
| `class` | `AstPlus` |
| `class` | `AstString` |
| `class` | `AstTrue` |
| `class` | `AstValue` |
| `class` | `BooleanNode` |
| `class` | `SimpleNode` |

| Fields in org.jdesktop.el.impl.parser declared as Node | |
| --- | --- |
| `protected  Node[]` | `SimpleNode.children` |
| `protected  Node` | `SimpleNode.parent` |

| Methods in org.jdesktop.el.impl.parser that return Node | |
| --- | --- |
| `Node` | `SimpleNode.jjtGetChild(int i)` |
| `Node` | `Node.jjtGetChild(int i)`             This method returns a child node. |
| `Node` | `SimpleNode.jjtGetParent()` |
| `Node` | `Node.jjtGetParent()` |
| `static Node` | `ELParser.parse(java.lang.String ref)` |

| Methods in org.jdesktop.el.impl.parser with parameters of type Node | |
| --- | --- |
| `void` | `SimpleNode.jjtAddChild(Node n, int i)` |
| `void` | `Node.jjtAddChild(Node n, int i)`             This method tells the node to add its argument to the node's list of children. |
| `void` | `SimpleNode.jjtSetParent(Node n)` |
| `void` | `Node.jjtSetParent(Node n)`             This pair of methods are used to inform the node of its parent. |
| `void` | `NodeVisitor.visit(Node node)` |

---


|  |  |  |
| --- | --- | --- |
| **Overview** \| **Package** \| **Class** \| **Use** \| **Tree** \| **Deprecated** \| **Index** \| **Help** | |  |
| PREV   NEXT | **FRAMES**    **NO FRAMES**     **All Classes** |  |


---
